# Dorsolateral medullary ischemic infarction causing autonomic dysfunction and headache: a case report

**DOI:** 10.1007/s10194-012-0427-8

**Published:** 2012-02-29

**Authors:** Riccardo Altavilla, Doriana Landi, Claudia Altamura, Gennaro Bussone, Paola Maggio, Marzia Corbetto, Federica Scrascia, Fabrizio Vernieri

**Affiliations:** 1 Neurologia Clinica, Università Campus Bio-Medico di Roma, Policlinico Universitario Campus Bio-Medico di Roma, Via Alvaro del Portillo 200, 00128 Rome, Italy; 2 Istituto Neurologico Carlo Besta, Milan, Italy

**Keywords:** Stroke, Secondary headache disorders, Autonomic diseases

## Abstract

Stroke can present, among other signs, with headache. Here, we describe the case of a man suffering from severe orbitary pain and autonomic dysfunction secondary to dorsolateral medullary ischemia. The anatomical relationship between lesion and symptomatology could be an indirect sign of hypothalamospinal tract involvement in the genesis of autonomic dysfunction and headache resembling a trigeminal autonomic cephalalgia.

## Introduction

Headache can be an accompanying symptom of cerebrovascular diseases (up to 38% of cases), mostly depending on stroke etiology and localization. It is very common in posterior inferior cerebellar artery infarction with a frequency up to 76% in patients with Wallenberg’s syndrome [1].

Few papers reported that headache resembling trigeminal autonomic cephalalgias (TACs) is induced by dorsolateral medullary ischemic infarction [2–4]. Here, we describe the case of a patient who developed headache and autonomic dysfunction after left dorsolateral medullary infarction.

## Case report

A 67-year-old Caucasian male was admitted to our hospital for left orbital, retro-orbital and temporal continuous pressure-like pain of moderate intensity, and gait disorder that suddenly started 15 days earlier. His medical history included hypertension, diabetes, myocardial infarction, cistectomy for bladder tumor, and an asymptomatic cerebral meningioma in left parietal area with the dimension of 20 × 16 mm, never surgically treated.

The patient was a heavy smoker. One month earlier, he arbitrarily stopped aspirin (100 mg/day) and ticlopidine (250 mg/day) intake.

Neurological examination revealed hypacusia on the left side, weaker corneal reflex, Horner’s syndrome, inferior facial hyposthenia and hypoesthesia, cold-like paresthesia in the first trigeminal branch territory, and no other signs of cranial nerves involvement or papillary edema. Muscular tone and strength were conserved; deep tendon reflexes were normal and symmetrical; signs of pyramidal tract involvement were absent. However, the patient showed limb ataxia with left lateropulsion, but co-ordination and other cerebellar functions were intact. Cranial CT scan and duplex ultrasound of cerebral and neck vessels were normal. Brain MRI revealed a small subacute left dorsolateral medullary infarction.

**Fig. 1:**
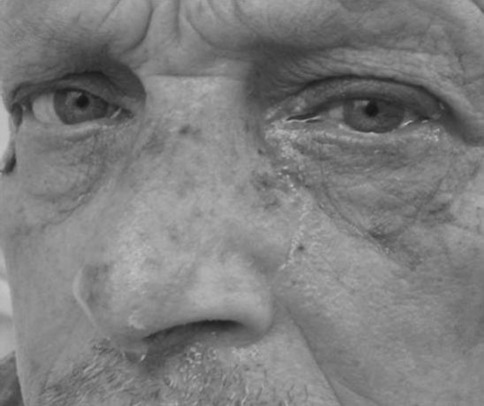
The figure shows patient’s lacrimation and conjunctival injection in the *left* eye associated with pain flairs

Few days later, the continuous pressure-like pain had resolved, but the patient experienced dramatic pain flares triggered exclusively when moving from clinostatic to orthostatic position, which he could hardly maintain. Pain exacerbations lasted as long as the patient stood and were associated with ipsilateral conjunctival injection, lacrimation, and nostril blockage (Fig. 1). Arterial blood pressure evaluation demonstrated orthostatic hypotension, changing from 150/90 mmHg in supine position to 115/80 mmHg in orthostatic position, persisting after 1, 3, and 5 min. These features were suggestive of autonomic dysfunction.

Common NSAIDs and pregabalin (150 mg/day) were ineffective in controlling pain. Indomethacin (100 mg/day), administered at the onset of pain for 3 days, slightly improved pain severity but not associated symptoms. However, it never prevented pain flares induced by standing up. Verapamil (240 mg/day) used on the basis of a previous report [3], successfully treated headache and vegetative phenomena but not Horner’s syndrome that partially recovered after 10 days.

At the 3-month follow-up visit, headache characteristics had changed since the subacute phase of stroke. Pain and associated symptoms clinically resembled TACs: attacks occurred several times a day (3–6), twice a week, for about 15–30 min, with milder pain than in subacute phase, and persistence of tearing and conjunctival injection. Orthostatic position did not trigger pain exacerbation any longer. Neurological examination showed left mild inferior facial palsy, dysesthesia in the first trigeminal branch territory, and ptosis; left miosis as well as orthostatic hypotension was no longer present. The patient was still on therapy with verapamil 240 mg/day.

The patient’s explicit and signed consent for publishing this case was obtained.

## Discussion

TACs are primary headaches characterized by typical pain and autonomic features [5]. Central nervous system lesions can rarely present with cluster-like or SUNCT-like symptomatology [2–4].

The hypothalamus, via the hypothalamospinal tract, is a regulatory centre for integration of sympathetic and parasympathetic systems. Experimental studies with functional MRI and PET showed hypothalamic activation during TACs attack [6]. Moreover, stereotactic hypothalamic stimulation has been successfully used in drug-resistant patients, indirectly confirming the hypothalamic involvement in TACs’ pathophysiology [7]. Hypothalamospinal tract lies in dorsolateral medulla; it is constituted by first order neurons responsible for orthosympathetic innervation of ipsilateral half face and body and projects to peri-acqueductal gray matter, thus activating the trigeminovascular system that is a well-known pain generator of headaches [8].

**Fig. 2:**
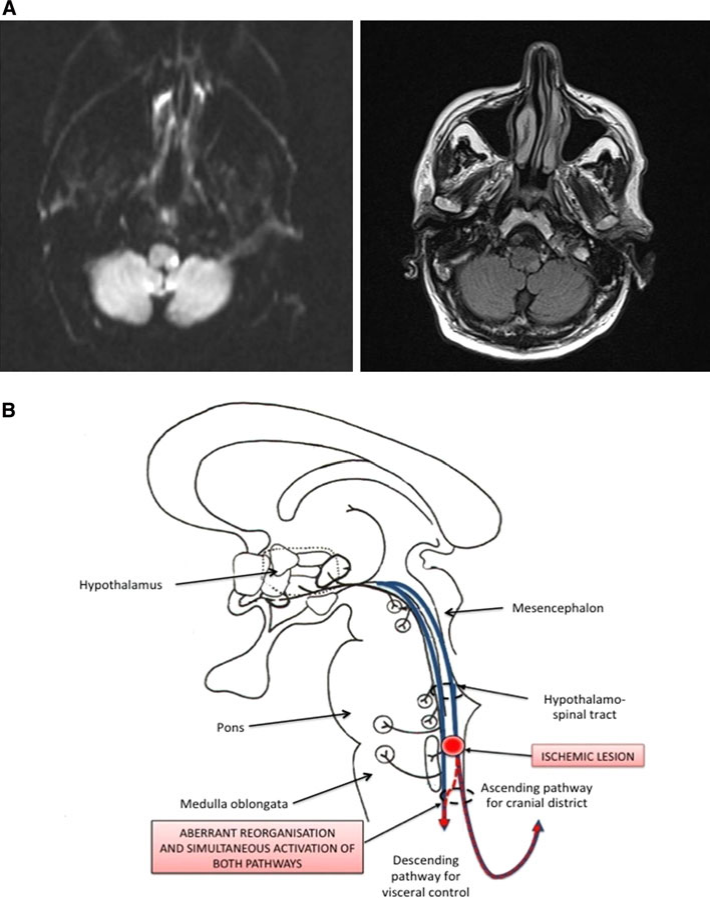
*Panel A*: diffusion weighted (*left*) and fluid attenuated inversion recovery (*right*) magnetic resonance showing the dorsolateral medullary ischemic infarction. *Panel B*: graphical representation of the hypothesized mechanism subtending our patient’s symptoms

In our patient, the ischemic lesion was located in the left posterior side of the upper medulla oblongata (Fig. 2a). In this area, descending fibers of the hypothalamospinal tract carry sympathetic innervation to the pericarotid plexus (Fig. 2b). In the subacute phase of stroke, Horner’s syndrome, as well as orthostatic hypotension, was symptomatic of a sympathetic impairment, while tearing and ocular injection reflected a parasympathetic activation. After 3 months, headache was still associated with vegetative symptoms but was no longer triggered by standing up, and lasted up to 30 min. In our opinion, the persistence of pain attacks with vegetative involvement in a chronic phase of stroke was due to an aberrant activation of trigeminovascular system by hypothalamospinal tract via the peri-acqueductal gray matter.

These clinical features resembled cluster headache. The weak response to indomethacin and the dramatic improvement after verapamil therapy supported this hypothesis. In fact, while indomethacin may be effective in treating paroxysmal hemicrania by inhibiting NO-induced dural vasodilation [9], verapamil acts mainly as neuromodulator in the hypothalamus [10].

This is the first case reporting the association of headache and orthostatic hypotension as part of an autonomic vascular impairment. In normal conditions, the hypothalamospinal tract is activated by standing up from a supine position via the baroreflex pathway so that the vasomotor reflexes lead to vasoconstriction and cardio-acceleration. We can speculate that in the subacute phase of stroke, the hypothalamospinal tract damage induces a dysfunction of sympathetic descending control of the peripheral vascular district (i.e., orthostatic hypotension) and an aberrant trigeminovascular hyperactivation via the peri-acqueductal gray matter (i.e., headache with vegetative symptoms). This phenomenon may be interpreted as maladaptive plasticity or as an effect of ephaptic connections. After 3 months, the evolvement of this maladaptive plastic phenomenon led to a more typical cluster-like headache.

In summary, our patient’s case supports the hypothesis of a dysfunction of the hypothalamospinal tract in the pathophysiology of both pain and autonomic features of TACs.
